# The Book World of Medicine and Science

**Published:** 1904-12-10

**Authors:** 


### The Book World of Medicine and Science.

The Surgery of the Diseases of the Appendix Vermiformis and their Complications. By W. H. Battle, F.R.C.S., and E. M. Corner, M.B., B.C., F.R.C.S. (London: Archibald Constable and Company, Limited. 1904. Pp. 208. Price 7s. 6d. net.)

So much has been written about the appendix, that the book under review cannot be otherwise than welcome, for it enables the busy practitioner to survey the present position of a department of medicine which has shown important advances in recent years. The authors are to be congratulated upon the comprehensive way in which they have dealt with a complicated subject in so small a compass. With the great majority of their statements there is no fault to find, although there are one or two inconsistencies noticeable which perhaps may be attributed to the dual authorship. For example, on page 60 we read that in acute appendicitis "it is very unusual for rigors to take place," whereas it is stated on page 149 that "a rigor is exceedingly rare in intestinal obstruction, whilst it is not uncommon in severe appendicitis." It is clear that one of these statements requires modification; and we should suggest that the former one unduly minimises the frequency of rigors in acute appendicitis. Another inconsistency is in the spelling. On one page we read "adhæsions" on the next "adhesion" appears, and as the word, spelt sometimes one way and sometimes the other, frequently recurs, the want of uniformity becomes very apparent. The meaning which it is intended to convey by the statement on page 59 that some cases of appendicitis "begin like a lion whilst they go out like a lamb" is very unfortunately expressed. On the same page the tongue is referred to as "the great index to the condition of the alimentary tract": a clinical definition which will certainly not meet with universal approval. On p. 69 there is an apparent commendation of ileostomy and appendicostomy in the treatment of colitis, methods of treatment that have proved more than disappointing in the hands of some who have tried them. We have made these criticisms, not with the intention of finding fault unnecessarily, but because we hope the faults will not appear in the second edition which we anticipate will be soon demanded; for in spite of its shortcomings the book is a good one, and we can recommend it to the profession.

International Clinics. Vol. III. Fourteenth Series. By various authors. Edited by A. O. J. Kelly, M.D., Philadelphia. (London: J. B. Lippincott Company. 1904. Pp. 302. Price 10s. 6d.)

The first half of the present volume is occupied by a series of articles on syphilis, all of which are instructive; but as we have no space to deal with each one separately, we shall briefly refer to one or two which are especially interesting. The first article is on uncertainty as to syphilitic inoculation, and is contributed by Mr. Campbell Williams. The author gives a lucid description of the atypical forms of early primary syphilitic lesions, and of the practical means of differentiating them from less nocuous conditions. He emphasises the necessity for delay in making a diagnosis where there is any doubt, and condemns entirely the practice of instituting mercurial treatment until the specific nature of the lesion is beyond doubt. Dr. Ohmann-Dumesnil contributes an excellent article on the differential diagnosis of syphilitic eruptions, and Dr. Spiller writes on syphilis of the nervous system. Dr. Fournier, of Paris, supplies material for reflection in a paper on syphilis and suicide. He classifies the cases in which suicide is attributable to syphilis into four groups: (1) Those due to the mental shock caused by the first discovery by a patient that he has the disease; (2) those caused by despair on account of serious syphilitic manifestations; (3) those connected with the social situation created by syphilis as regards marriage; and (4) those in which the suicide is the result of mental disorder produced by syphilis. Dr. Fournier graphically describes one or two instances which have come to his knowledge, and advises that caution be exercised in telling a patient the true condition of affairs. The information is often best conveyed in the form of a suspicion ripening into certainty in the course of a few days.

The second half of the book is composed of sections applied to treatment, medicine, surgery, gynæcology, and neurology. All the papers are good, and we can cordially recommend the present volume, like its predecessors, to the notice of the medical profession.

Health in Infancy. By T. M. Allison, M.D. (London: Simpkin, Marshall, Hamilton, Kent, and Company. Pp. 41. 10 Illustrations. Price 1s. net.)

Dr. Allison's booklet may be fairly described as a piece of special pleading for natural—that is, breast feeding. That the experience of the medical profession is in complete agreement with that of the author on this matter needs no reiteration, but he makes an appeal in simple yet forcible language which is well worthy of consideration by the public. Dr. Allison well says that what is wanted for the race is not strength only, but staying power, and he has undoubtedly pointed out one of the main conditions for its attainment. A novel feature is the introduction of photographs not only of infants, but of foals and calves, to illustrate the difference of development attained when natural as opposed to artificial feeding is practised. The former have the advantage not only in size, but have an appearance of healthy animation conspicuously absent in those fed by hand.

Practical Advertising, 1904–1905. (London: Mather and Crowther.)

The new issue of this work is prefaced by an article of interest to advertisers, which is followed by a series of beautifully printed designs in colours illustrating some of the posters adopted by the leading advertisers. The lists and particulars of the newspapers and periodicals published in the United Kingdom and the colonies appear to be very complete and up-to-date. No trouble evidently has been spared in the production, and the book is an excellent example of the perfection to which printing has been brought within the last few years.

